# 700,000 years of tropical Andean glaciation

**DOI:** 10.1038/s41586-022-04873-0

**Published:** 2022-07-13

**Authors:** D. T. Rodbell, R. G. Hatfield, M. B. Abbott, C. Y. Chen, A. Woods, J. S. Stoner, D. McGee, P. M. Tapia, M. Bush, B. L. Valero-Garcés, S. B. Lehmann, S. Z. Mark, N. C. Weidhaas, A. L. Hillman, D. J. Larsen, G. Delgado, S. A. Katz, K. E. Solada, A. E. Morey, M. Finkenbinder, B. Valencia, A. Rozas-Davila, N. Wattrus, S. M. Colman, M. G. Bustamante, J. Kück, S. Pierdominici

**Affiliations:** 1 Geoscience Department, Union College, Schenectady, NY USA; 2 Department of Geological Sciences, University of Florida, Gainesville, FL USA; 3 Department of Geology and Environmental Science, University of Pittsburgh, Pittsburgh, PA USA; 4 Department of Earth, Atmospheric and Planetary Sciences, Massachusetts Institute of Technology, Cambridge, MA USA; 5 Nuclear and Chemical Sciences Division, Lawrence Livermore National Laboratory, Livermore, CA USA; 6 College of Earth, Ocean, and Atmospheric Sciences, Oregon State University, Corvallis, OR USA; 7 Instituto Nacional de Investigación en Glaciares y Ecosistemas de Montaña, Huaraz, Perú; 8 Institute for Global Ecology, Florida Institute of Technology, Melbourne, FL USA; 9 Instituto Pirenaico de Ecología, Consejo Superior de Investigaciones Científicas, Zaragoza, Spain; 10 Department of Atmospheric and Environmental Sciences, University at Albany, State University of New York, Albany, NY USA; 11 Department of Geology, Occidental College, Los Angeles, CA USA; 12 Department of Earth and Environmental Sciences, University of Michigan, Ann Arbor, MI USA; 13 Department of Environmental Engineering and Earth Sciences, Wilkes University, Wilkes-Barre, PA USA; 14 Ciencias de la Tierra y Clima, Universidad Regional Amazónica Ikiam, Tena, Ecuador; 15 Department of Earth and Environmental Sciences, University of Minnesota Duluth, Duluth, MN USA; 16 Large Lakes Observatory, University of Minnesota Duluth, Duluth, MN USA; 17 Helmholtz-Zentrum Potsdam, Deutsches GeoForschungsZentrum GFZ, Potsdam, Germany

**Keywords:** Palaeoclimate, Limnology

## Abstract

Our understanding of the climatic teleconnections that drove ice-age cycles has been limited by a paucity of well-dated tropical records of glaciation that span several glacial–interglacial intervals. Glacial deposits offer discrete snapshots of glacier extent but cannot provide the continuous records required for detailed interhemispheric comparisons. By contrast, lakes located within glaciated catchments can provide continuous archives of upstream glacial activity, but few such records extend beyond the last glacial cycle. Here a piston core from Lake Junín in the uppermost Amazon basin provides the first, to our knowledge, continuous, independently dated archive of tropical glaciation spanning 700,000 years. We find that tropical glaciers tracked changes in global ice volume and followed a clear approximately 100,000-year periodicity. An enhancement in the extent of tropical Andean glaciers relative to global ice volume occurred between 200,000 and 400,000 years ago, during sustained intervals of regionally elevated hydrologic balance that modified the regular approximately 23,000-year pacing of monsoon-driven precipitation. Millennial-scale variations in the extent of tropical Andean glaciers during the last glacial cycle were driven by variations in regional monsoon strength that were linked to temperature perturbations in Greenland ice cores^1^; these interhemispheric connections may have existed during previous glacial cycles.

## Abstract (web summary)

Analysis of a continuous and independently dated record of glaciation in the tropical Andes spanning 700,000 years shows that Andean glaciation follows patterns of global ice volume change, with a periodicity of approximately 100,000 years.

## Main

The δ^18^O record of marine benthic foraminifera^2,3^ (LR04) has provided the foundational framework for our understanding of late Cenozoic ice ages. However, because about 80% of global ice volume change over glacial–interglacial cycles occurred in the middle to high latitudes of the Northern Hemisphere (NH)^4,5^, the marine δ^18^O record tells us little about the timing and extent of glaciation in the Southern Hemisphere (SH) and in all tropical mountain ranges. Radiometric dating of moraines in the SH^6,7^ confirms that the local last glacial maximum occurred roughly at the same time as the last peak of global ice sheet volume (marine isotope stage 2; MIS 2). However, moraine records are inherently discontinuous, with younger glacial advances commonly obliterating evidence of older ice positions, making it challenging to make interregional comparisons of the timing of ice build-up and decay over several ice-age cycles using moraine chronologies alone. By contrast, lake sediment records can provide continuous archivesof up-valley glaciation^8^, but long lacustrine records of tropical glaciation have thus far been limited by a lack of sufficient independent age control and/or a lack of clarity about the glacial signal preserved^9,10^.

Here we report an approximately 700 thousand year ago (ka) continuous and independently dated lacustrine record of alpine glaciation from the central Peruvian Andes that is directly comparable with records of extratropical temperature change, global ice volume and atmospheric greenhouse gas (GHG) concentrations. We show that tropical Andean glaciers waxed and waned with the NH on orbital timescales but that the relative amplitude of glacial–interglacial cycles has not been constant. During much of MIS 7–11, tropical Andean glacier extents were enhanced relative to global ice volume, and this enhancement may have been related to increased precipitation coupled with reductions in the concentration of atmospheric CH_4_ (ref. ^11^). Millennial-scale perturbations in Andean glacier extent during the last glacial cycle were similar in timing to both regional cave records of precipitation and to temperature oscillations recorded in Greenland ice cores^1^; this pattern of change may have also occurred during previous glacial cycles. Thus, persistent multiscaled interhemispheric climatic teleconnections affected tropical Andean glaciers during much of the past 700 ka.

## Lake setting and glaciation

**Fig. 1:**
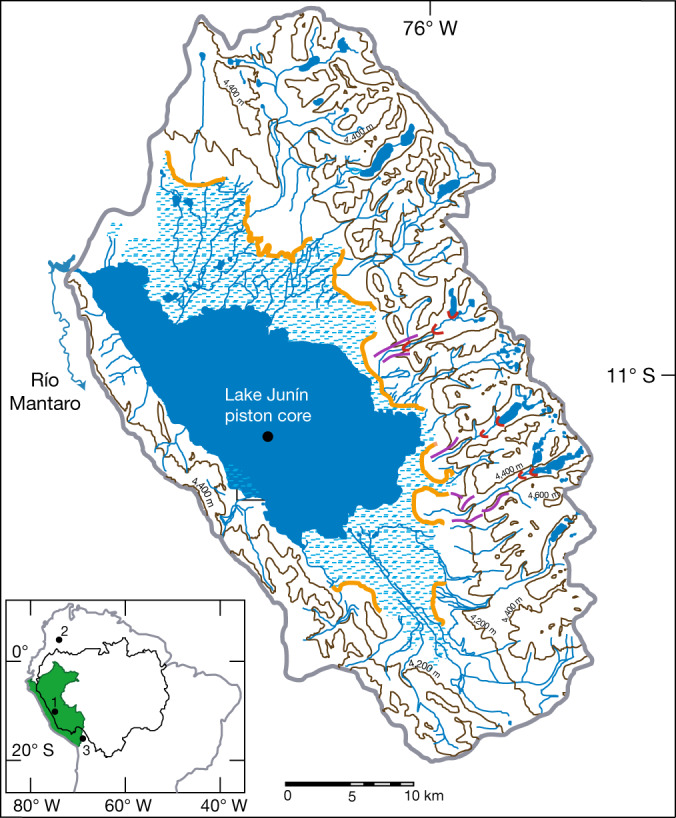
Location of Lake Junín and drainage basin in the central Peruvian Andes. Contour interval 200 m; stippled blue pattern indicates wetlands; orange arcuate lines are toes of proglacial outwash fans; red arcuate lines are ^10^Be-dated MIS 2 and 3 moraines^6^; purple lines are ^10^Be-dated pre-MIS 3 moraines^6^; grey outline is the drainage basin (see Methods). Inset map: black line demarcates the Amazon drainage basin; solid green fill is Perú;  black circles indicate Andean records discussed in the text: 1, Lake Junín, Perú; 2, Sabana de Bogotá^9^; 3, Lake Titicaca^10^.

Lake Junín (11° S, 76° W, 4,100 metres above sea level (masl)) is a seasonally closed-basin lake located in the uppermost Amazon basin between the eastern and western cordilleras of the Peruvian Andes (Fig. 1). Glacial outwash fans and lateral moraines form the eastern and northern edges of the basin (Fig. 1), and ^10^Be exposure ages from these moraines indicate that they span several glacial cycles^6^, but at no time during the past 700 ka has the lake been overridden by glacial ice. Thus, Lake Junín is ideally situated to record cycles of tropical glaciation^12^. During the local last glacial maximum, alpine glaciers descended from cirque headwalls at about 4,700 masl to moraine positions about 4,160 masl, within several kilometres of the modern lake shoreline^6^. A seismic reflection survey (see Methods) identified a main reflector at a depth of about 100 m below lake floor, which corresponds to the depth of the base of the composite section of lacustrine sediment (about 95 m) reported here. This reflector represents a change in sedimentation, from fluvial sand and gravel to well-sorted silt and clay. This transition, dated to MIS 16, documents the creation of Lake Junín by coalescing glacial outwash fans and the onset of continuous lacustrine sedimentation.

**Fig. 2:**
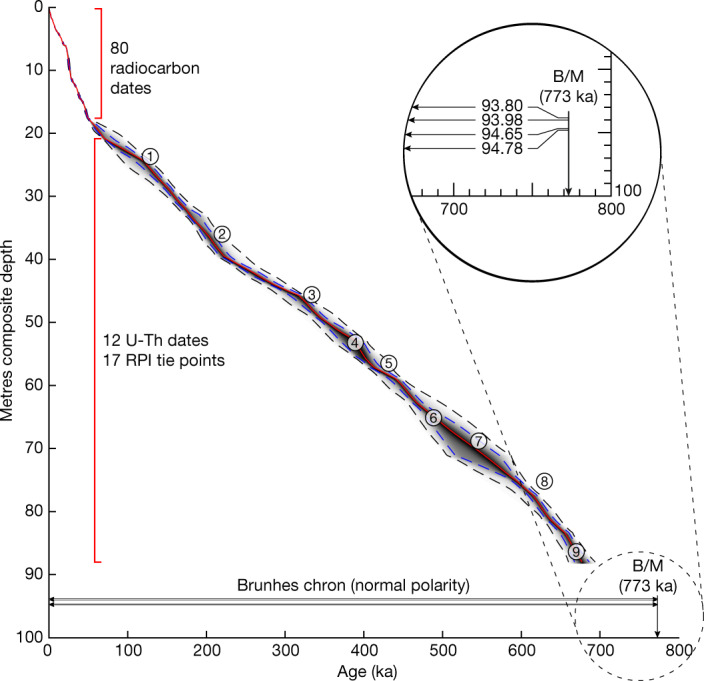
Age–depth model for the Junín piston core. The age–depth relationship is based on 80 radiocarbon dates^1^ (<17 metres composite depth (mcd)), 12 U-Th-dated intervals of authigenic calcite from five carbonate intervals between about 21 and 71 mcd (ref. ^14^) and 17 palaeomagnetic tie points^15^. The red line is the mean age model; purple and black dashes represent the 1 sigma and 2 sigma uncertainties around the mean, respectively. Four arrows mark the depth of four samples that yielded normal polarity (depths shown in the inset along the age of the Brunhes–Matuyama (B/M) reversal boundary) and are younger than 773 ka (ref. ^16^) (see Methods). Numbers 1–9 are tie points (Fig. 3b,c) used as the age model for Fig. 4a–c; tie points are for illustration only and were not used in the generation of this radiometric and palaeomagnetic age model^15^. Source data

**Fig. 3:**
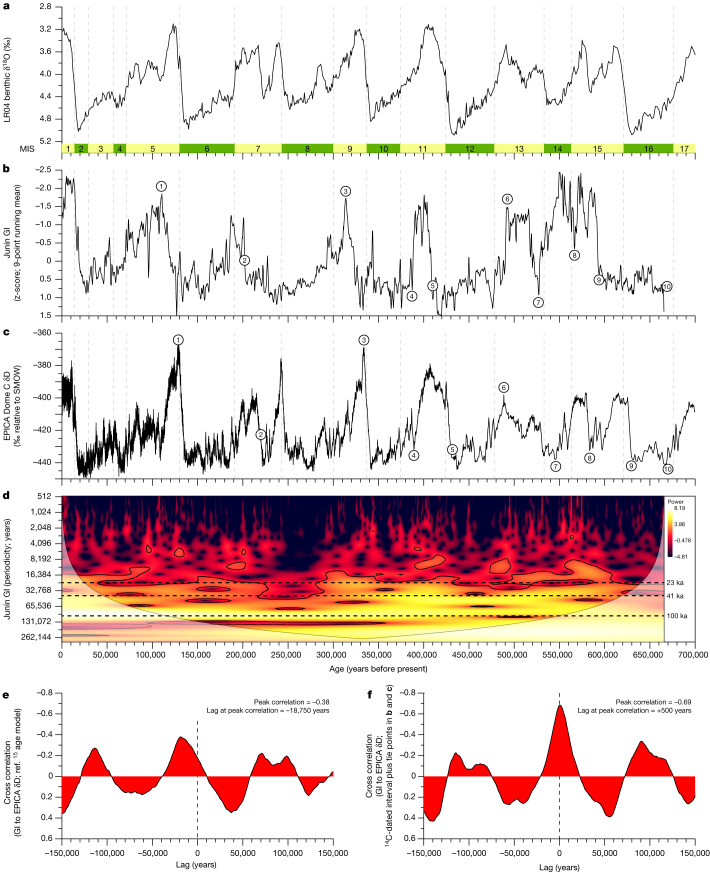
Comparison of the Junín record of tropical Andean glaciation with extratropical records of glaciation and temperature change. **a**–**f**, Benthic δ^18^O proxy of global ice volume^3^ and MIS boundaries (**a**), Junín GI based on the radiometric and palaeomagnetic age model^15^ (**b**), EPICA Dome (Antarctica) δD temperature proxy^18^ (**c**), wavelet analysis^37^ of the GI record based on the radiometric and palaeomagnetic age model^15^ (**d**), and cross-correlation plots of **b** and **c** using the radiometric and palaeomagnetic age model^15^ (**e**) and the age model based on 80 radiocarbon ages^1^ and tie points in **b** and **c** and circled in Fig. 2 (**f**). SMOW, standard mean ocean water. Source data

Piston cores with a composite length of about 95 m (ref. ^13^) were extracted from the lake’s depocentre (Fig. 1) as part of the International Continental Scientific Drilling Program (ICDP). The age–depth model of the upper 88 m was established with 80 accelerator mass spectrometry ^14^C ages from the upper 17 m (ref. ^1^), 12 U-Th-dated intervals of authigenic calcite from five carbonate intervals between about 21 and 71 m (ref. ^14^) and 17 geomagnetic relative palaeointensity (RPI) tie points between 24 and 88 m (ref. ^15^) (Fig. 2), yielding an age of 677 ± 20 ka at 88 m. Four samples from near the base of the lacustrine section (93–95 m) show normal-polarity palaeomagnetic directions (see Methods) (Fig. 2), consistent with sediment deposition during the Brunhes chron and a basal age younger than 773 ka (ref. ^16^). As the age model is not orbitally tuned, the Lake Junín record enables an independent comparison of the pacing of tropical glaciation with that of LR04 (ref. ^3^), which is a proxy for global ice volume and benthic ocean temperature (Fig. 3a).

**Extended Data Fig. 1:**
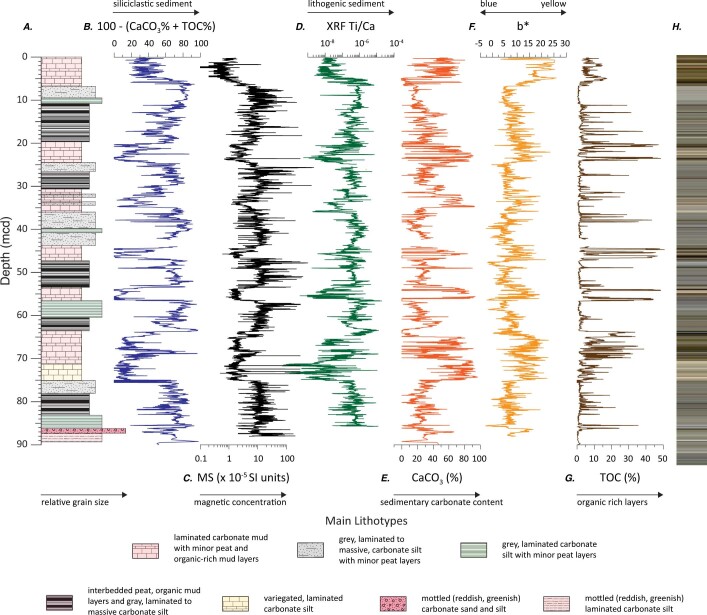
Lithostratigraphy and physical properties of the Junín piston core. Stratigraphic column (**a**) of main lithotypes in the drill core from the depocentre of Lake Junín and downcore plots illustrating variations in percentage siliciclastic sediment (**b**), magnetic susceptibility (MS; **c**), Ti/Ca (**d**), CaCO_3_ (**e**), b* sediment colour index (**f**), percentage total organic carbon (TOC; **g**) and digital composite core image (**h**). Source data

**Extended Data Fig. 2:**
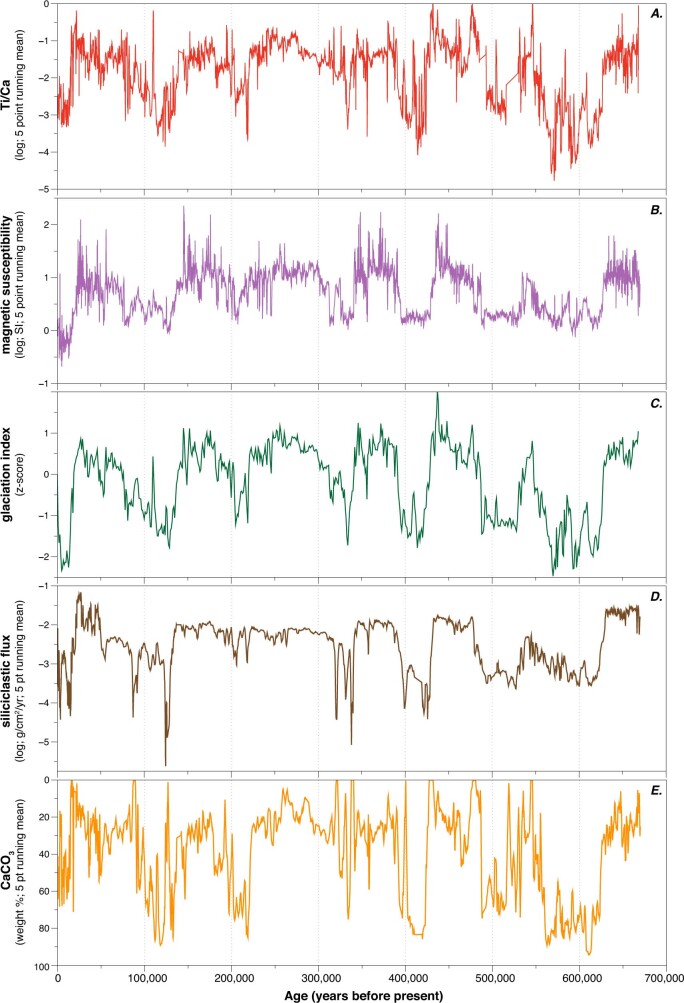
Proxy indicators of clastic sediment concentration in the Lake Junín piston core. Data are plotted on the age model tuned to EPICA Dome (Antarctica) δD temperature proxy^18^ (Fig. 4a). Ti/Ca (**a**), magnetic susceptibility (**b**), Junín GI (see Methods; **c**), siliciclastic sediment flux (**d**) and carbonate content (**e**). Source data

The composite section comprises intervals of siliciclastic sediment intercalated with intervals dominated by authigenic calcite (about 60–90%; Extended Data Fig. 1). The siliciclastic-rich intervals have a consistent signature, with relatively low concentrations of carbonate (<40%) and organic carbon (<5%), and high values of bulk density (>1.3 g cm^−3^), magnetic susceptibility (MS) and concentrations of elements (such as Ti, Si and K) derived from erosion of the non-carbonate fraction of the regional bedrock (Extended Data Fig. 1). The Junín glaciation index (GI; Fig. 3b, Methods and Extended Data Fig. 2) integrates MS and Ti/Ca, and is a proxy for clastic sediment flux and the extent of regional ice cover. During MIS 2 and 3, regional snowlines were around 300–600 m lower than modern estimates^17^ and ^10^Be-dated moraines^6^ confirm expanded ice cover in the valleys to the east of Lake Junín (Fig. 1). During this interval, the GI was elevated and siliciclastic flux to Lake Junín was 1–2 orders of magnitude higher than during the subsequent late glacial and Holocene^1,8^ (Fig. 3b and Extended Data Fig. 2). Together, these observations (see Methods and Extended Data Fig. 2) link variations in the GI with changes in regional ice extent.

### Comparison with global ice volume

The Lake Junín GI record over the past 677 ka shows a strong similarity to LR04 (ref. ^3^). Seven glacial–interglacial cycles are evident in the GI and, within the uncertainty of the age model^14,15^, they mimic changes in LR04 and in the δD of Antarctic ice^18^, a proxy for high-latitude SH air temperature (Fig. 3a–c). The 100-ka cycle is the strongest and most pervasive orbital periodicity observed (Fig. 3d). The basal age between about 677 and 773 ka indicates that damming of the Junín basin by proglacial deposits occurred near the end of the middle Pleistocene transition^19^, when global ice volume increased and began to beat to a 100-kyr rhythm. We propose that, before this transition, tropical glaciers were too limited in extent to generate the extensive outwash fans needed to dam the intermontane basin that houses the lake today. After this transition, tropical glaciers clearly followed the global 100-kyr beat, in sync with the rhythm of Andean forest dynamics documented in the Sabana de Bogotá record^9^. The presence of the 41-kyr orbital band may reflect the direct role of obliquity in tropical hydroclimate^20^ and/or the role of obliquity in global ice volume and GHG concentrations that, in turn, drive tropical climate change. The 23-kyr precessional band has been previously recognized as a key orbital pacer of the South American summer monsoon (SASM) and the hydrologic balance of the upper Amazon basin and Altiplano^12,21^. Given the orders-of-magnitude difference in response times between alpine glaciers and NH ice sheets, the close match between the GI and LR04 (Fig. 3a–c) requires that the former was forced by the latter, rather than directly by the underlying orbital parameters behind NH ice sheet growth, namely, low boreal summer insolation. Transient climate model experiments^22^ conclude that GHGs explain this relationship during the last deglaciation: the increase in CO_2_ and CH_4_ forced a synchronous retreat of glaciers globally in spite of hemispheric and latitudinal heterogeneities in regional insolation^22^. Assuming that GHGs were also responsible for the growth as well as the decay of tropical glaciers throughout the late Quaternary, the mechanism(s)^23^ linking orbital forcing, NH ice sheets, GHGs and tropical glaciation must have been relatively rapid, as evidenced by the lack of measurable lag time in tropical ice core records of the last deglacial temperature rise^24^.

### Amplitude of glacial cycles

**Fig. 4:**
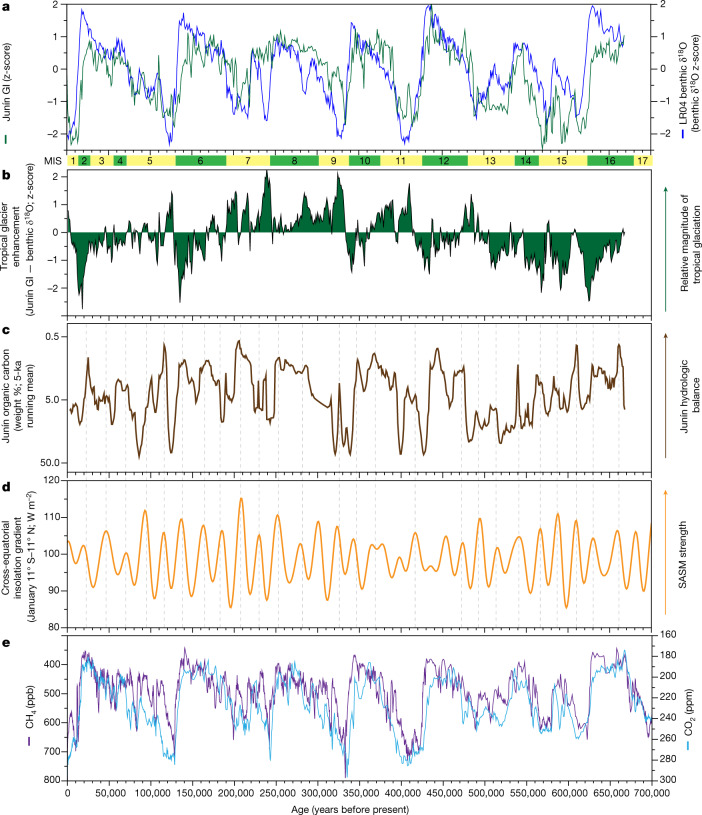
Comparison of the amplitude of glacial cycles in the tropical Andes and global ice volume. **a**–**e**, Junín GI and benthic δ^18^O proxy of global ice volume^3^ (z-scores) using 80 radiocarbon ages^1^ and tie points in Figs. 2 and 3b,c for the Junín GI age model (**a**), index of tropical glacier enhancement from the difference between the Junín GI and the LR04 proxy of global ice volume^3^ (**b**), weight percentage organic carbon in Junín drill core (**c**), cross-equatorial January insolation gradient^38^ between 11° S and 11° N (**d**) and atmospheric GHG concentrations from Antarctic ice cores (**e**; CO_2_ (refs. ^39,40^); CH_4_ (ref. ^41^)). Dashed vertical lines in **c** and **d** highlight the tight coupling between the Junín lake level and the regional monsoon strength. Source data

**Extended Data Fig. 3:**
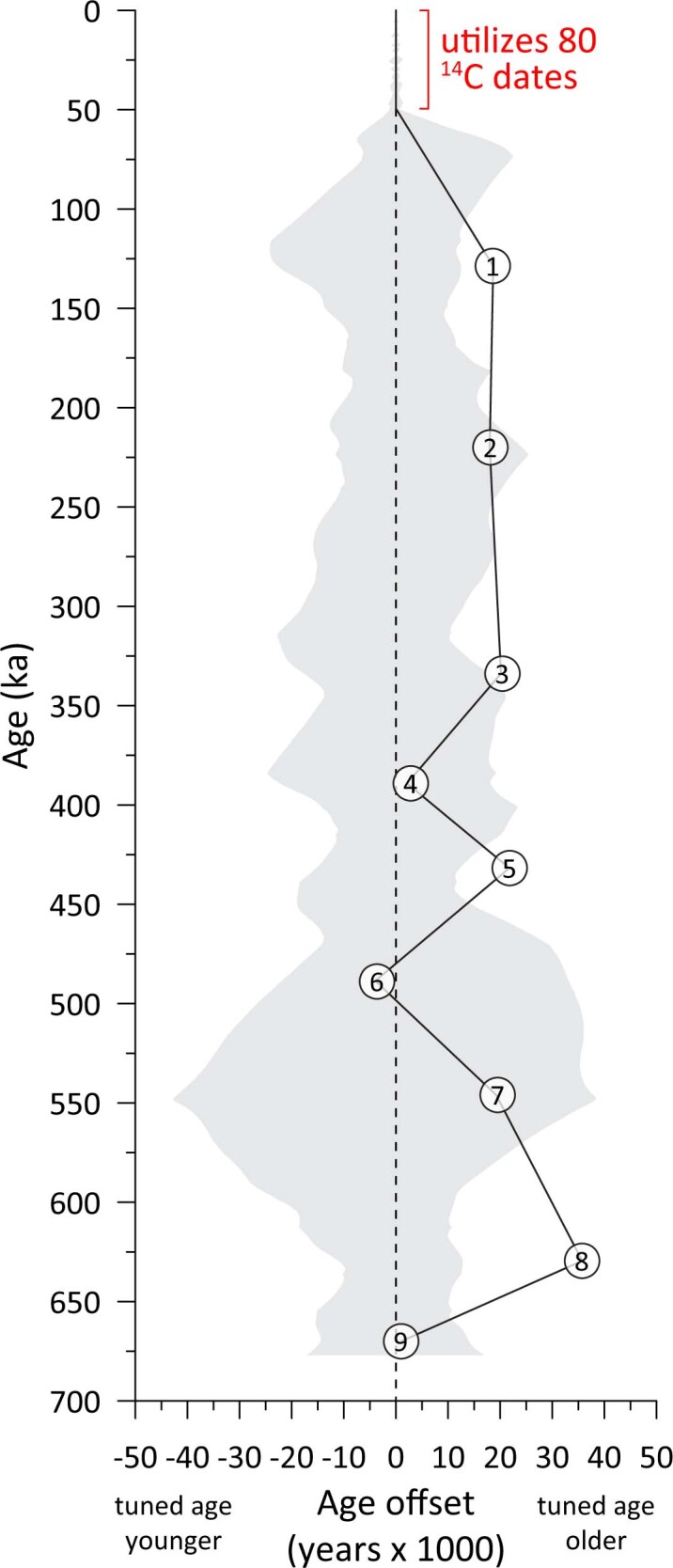
Tuning points applied to the radiometric age model for the Junín piston core. Age offset between the age model derived by tuning Junín GI to EPICA Dome (Antarctica) δD temperature proxy^18^ and the radiometric and RPI-based age model^15^ (black line) using the nine tie points in Figs. 2 and 3 (numbers). Positive (negative) values denote that the tuned age is older (younger) than the radiometric and RPI-based age model, with the differences largely falling within ±2 sigma uncertainty of the radiometric and RPI-based age model^15^ (grey shading). Source data

To compare the relative extent of tropical glacial cycles and global ice volume over the past 700 ka, we synchronized the Junín GI (Fig. 3b) and Antarctic (EPICA) δD (ref. ^18^) records using nine tie points (Figs. 2 and 3b,c) that correlate prominent peaks and troughs in both records; these nine tie points supplement 80 radiocarbon dates from the upper 18 m of the Junín core^1^ (Fig. 2). Seven of the nine tie points are within the 2 sigma uncertainty of the radiometric and RPI age model^15^ (Fig. 2 and Extended Data Fig. 3). Synchronizing the main features of the Junín GI record with EPICA δD (Fig. 4a) reduces the lag between the Junín GI and EPICA δD from −18,750 years to +500 years (Fig. 3e,f). The systematic 18,750-year lag in the Junín GI record relative to EPICA δD (Fig. 3b,c) may be an artefact of the limitation of U-Th to date only the low sedimentation rate, high carbonate (interglacial) intervals and not the glacial intervals that are marked by higher sedimentation rates and low authigenic carbonate.

Comparison of the relative amplitude of the z-score Junín GI and LR04 records on the EPICA δD resolved chronology shows clear similarities and differences (Fig. 4a). The correlation coefficient (*r*) for the Junín GI and LR04 for the full record is 0.62; for the past 140 ka, it is 0.74. The similarity between the records for the MIS 1–6 window does not support the conclusion^25^ that MIS 4 was an anomalously large glacial cycle based on cosmogenic radionuclide ages on moraines from the middle and low latitudes of both hemispheres. The index of tropical glacier enhancement (TGE; Fig. 4b) quantifies the degree to which the Junín GI exceeds (positive values) or falls below (negative values) z-score normalized changes in LR04. Long-term (about 10^4^–10^5^-year) variations in TGE may be driven by differences in tropical versus global ice volume and/or by long-term changes in basin sensitivity to record clastic sediment, such as through the progressive progradation of glacial outwash fans (Fig. 1 and Methods). However, unlike climatic forcings, long-period changes in basin ontogeny are probably unidirectional, thus we interpret long-term cycles of TGE to reflect relative differences in the magnitude of glaciation between the tropical Andes and LR04. There seems to be an approximately 400-kyr-long variability in TGE, with the interval from 200 ka to 400 ka (MIS 7–11) marked by elevated TGE (Fig. 4b). High GI and TGE during this window suggest that glaciers maintained some presence in the Junín catchment during the interglacial intervals of MIS 7, 9 and 11, when global ice volume was generally low. Retention of glacial ice during MIS 7, 9 and 11 is reflected in the millennial signature of the GI during parts of these three interglacial intervals (Fig. 3d). Organic-rich sediment and fibrous peat are the dominant sediment facies during intervals of low lake level^1^; carbonate ‘marl’ and/or glacial flour dominate intervals of elevated lake level (Extended Data Figs. 1 and 2). Accordingly, the 200–400-ka interval of elevated TGE comprises extended periods of elevated hydrologic balance (lake level) in Lake Junín, as reflected by minima in organic carbon (Fig. 4c). The level of Lake Junín (Fig. 4c) is tightly coupled to the cross-equatorial insolation gradient (Fig. 4d), which today is associated with SASM strength. Variability in the cross-equatorial insolation gradient clearly follows the 23-kyr beat of Earth’s precession and is modulated on a 400-kyr cycle, which reflects Earth’s eccentricity. The tight coupling between the level of Junín and the SASM (vertical dashed lines in Fig. 4c,d) seems to break down during the interval of elevated TGE (about 200–400 ka); during this latter window, the amplitude of hydrologic variability at Junín was reduced and several longer period (50–75-kyr) intervals of elevated hydrologic balance occurred. Perhaps non-SASM-driven precipitation supplemented monsoonal precipitation during this interval. Whatever the cause, heightened precipitation apparently maintained small alpine glaciers in the valleys to the east of Lake Junín, especially during intervals of reduced CH_4_ (ref. ^11^) (Fig. 4e).

### Millennial events

**Extended Data Fig. 4:**
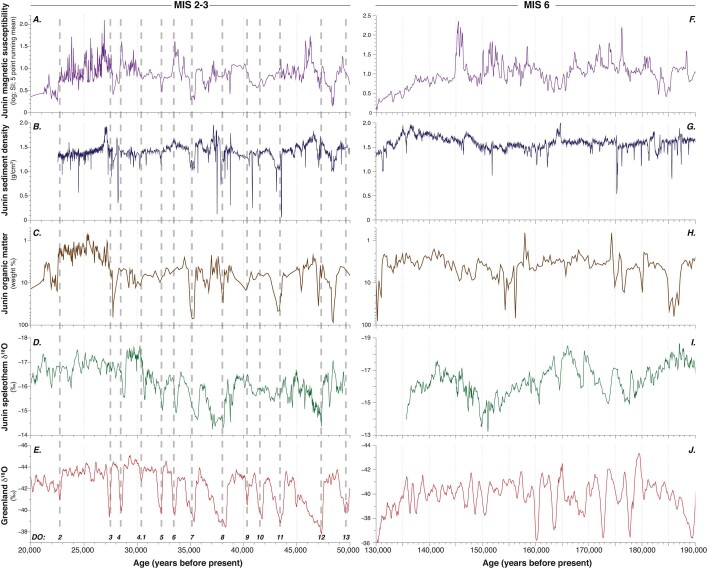
Comparison of millennial-scale variability in the Junín piston core with regional cave records of hydroclimate and Greenland air temperature during MIS 2, 3 and 6. High-resolution MS is a proxy for siliciclastic flux and regional ice extent (see Methods and Extended Data Fig. 2) and organic matter content is an inverse proxy for the hydrologic balance of Lake Junín^1^. **a**–**e**, Expanded views of the 20–50-ka (MIS 2 and 3) interval. **f**–**j**, Expanded views of the 130–190-ka (MIS 6) window. **d**, Pacupahuain Cave (Fig. 1) speleothem record^26^. **e**, Greenland δ^18^O record^49^. **i**, Huagapo Cave (Fig. 1) speleothem record^31^. **j**, Synthetic Greenland δ^18^O record^32^. Dashed lines in **a**–**e** illustrate the temporal connection between Dansgaard–Oeschger (DO) events in Greenland ice cores and intervals of drying and deglaciation in the tropical Andes. Source data

The last glacial cycle in the Junín region was punctuated by intervals of abrupt glacial retreat, as evidenced by lowered values of Ti, MS and sediment density, and lowered lake level, as recorded by the accumulation of organic-rich sediment in the centre of the Junín basin^1^ (Extended Data Fig. 4). The abrupt decreases in glacial erosion and lake level correlate^1^ with increases in the δ^18^O record from nearby (about 20 km south-east of Lake Junín) Pacupahuain Cave^26^ and also with the warming episodes recorded in Greenland ice cores, known as Dansgaard–Oeschger (DO) events^1,27^ (Extended Data Fig. 4). The synchronicity among DO events and retreating glaciers in the Junín watershed document a climatic teleconnection linking circum-North Atlantic temperatures with the mass balance of glaciers in the upper Amazon basin during the last glacial cycle^1^. Abrupt DO warmings in Greenland resulted in a northward shift in the mean position of the intertropical convergence zone, which weakened the SASM and reduced moisture advection to the eastern Andes and South American Altiplano^1,26,28^. Thus, the millennial-scale fluctuations in the extent of glaciers in the tropical Andes may have been driven by changes in precipitation (snowfall) amount in response to instabilities in North Atlantic temperatures. This is consistent with established linkages between North Atlantic climate and the hydrologic balance of Altiplano lakes^21^. Whereas glaciers in the inner tropics of the Andes are especially sensitive to temperature because of sustained precipitation year-round, glaciers in the outer tropics, such as those at the latitude of the Junín basin, experience greater seasonality of precipitation and are twice as sensitive to changes in precipitation as those in the inner tropics^29,30^.

Millennial-scale instabilities may have also affected the tropical Andes during previous glacial cycles, although age–depth model uncertainty (±5–10%) in the Junín core beyond 50 ka (Fig. 2) precludes precise correlation among records. During the penultimate glacial cycle (MIS 6), a similarity in the pattern of variability between a speleothem δ^18^O record from nearby (about 25 km south-east of Lake Junín) Huagapo Cave^31^ and a synthetic Greenland δ^18^O record for MIS 6 (ref. ^32^) further suggests long-term forcing of the hydrologic balance and, thus, glacier extents in the SH tropics by instabilities in North Atlantic climate^31^ (Extended Data Fig. 4). However, the strong role of North Atlantic climatic instability on tropical Andean hydrologic and palaeoglacier mass balance may not have extended through all glacial terminations. For example, during the waning stages of MIS 2, a prominent glacial readvance in the higher elevations of the tropical Andes seems to have occurred during the Antarctic Cold Reversal (approximately 14.5–12.9 ka)^33^. The Junín GI does not record this event, however, because the elevation of the Junín catchment is too low to have supported glacial ice at that time^6^.

This continuous, independently dated record of tropical glaciation confirms the role of fast-acting teleconnections, many apparently originating in the high latitudes of the NH over the past 700 ka. Global GHGs, modulated by orbitally forced NH ice sheet growth^34^, drove atmospheric temperatures in the tropics over ice-age cycles, whereas instabilities in North Atlantic climate and monsoon precipitation acted as secondary effects on tropical ice extent. The mass balance of the small alpine glaciers of the tropical Andes rapidly integrated the effects of both air temperature and moisture flux with short response times^35^. Because mid-tropospheric tropical temperature is nearly uniform with longitude owing to weak Coriolis force at low latitudes^36^, this record may be broadly representative of tropical glaciers around the world over the past 700 ka.

## Methods

### Location map

The topography of the Junín basin (Fig. 1) is based on four 1:100,000-scale topographic maps published by the Instituto Geográfico Nacional^42–45^.

### Seismic reflection survey

High-resolution (4–24-kHz), shallow-penetration data were collected in 2008 and an air gun survey was conducted in 2011 (ref. ^46^). The air gun seismic survey imaged over 100 m of nearly horizontal sediment with no evidence of deformation. Details of the stratigraphy were obscured by ringing in the shallow water column, but several prominent, continuous reflectors were observed that could be traced across the lake basin. One of these, at a depth of approximately 100 m below the lake floor, was especially prominent and could be mapped throughout the survey area and was noted to be flatter than the modern lake floor. This suggests broad-scale basin subsidence and a lack of local tectonic deformation. Drill cores showed that this reflector marks a transition from fluvial sand and gravel to finer-grained lacustrine sediment. At the time this deep reflector was formed, the lake was apparently larger than at present. No evidence was observed in the central part of the lake for large-scale lake-level fluctuations, such as wave-cut shorelines, fluvial channels or low-stand deltas.

### Core analyses

Sediment density and MS were measured every 0.5 cm at the National Lacustrine Core Facility (LacCore) at the University of Minnesota (MN, USA). Density was measured on the whole-round cores by gamma ray attenuation using a multisensor core logger and MS was measured by a Bartington MS2E point sensor on the split core surface. Furthermore, density was measured from the air-dried mass of 1-cc samples taken every 2.5 cm above 6.665 m and every 8 cm below 6.665 m. X-ray fluorescence (XRF) scanning was performed at the LacCore XRF Lab, University of Minnesota Duluth (MN, USA) Large Lakes Observatory using a Cox Analytical ITRAX with a Cr tube, 5-mm resolution and 15-s dwell time. Total organic and inorganic carbon (TOC and TIC, respectively) was measured on samples taken every 2.5 cm above 6.665 m and every 4 cm below 6.665 m. Total carbon was determined by combusting samples at 1,000 °C using a UIC CM5200 automated furnace and analysing the resultant CO_2_ using a UIC CM5014 coulometer at the Sediment Core Laboratory at Union College (NY, USA). Similarly, TIC was determined by acidifying samples using an Automate acidification module and measuring the resultant CO_2_ by coulometry. We calculated the weight percentage TOC from TOC = TC − TIC. We measured the biogenic silica (bSiO_2_) content of a total of 65 samples obtained randomly from all facies present in the sediment core following the procedure outlined in ref. ^47^. Siliciclastic flux (Flux_clastic_) was calculated as:

$${{\rm{F}}{\rm{l}}{\rm{u}}{\rm{x}}}_{{\rm{c}}{\rm{l}}{\rm{a}}{\rm{s}}{\rm{t}}{\rm{i}}{\rm{c}}}={\rm{S}}{\rm{R}}\times ({\rm{B}}{\rm{D}}-(({\rm{B}}{\rm{D}}\times {\rm{T}}{\rm{O}}{\rm{M}})+({\rm{B}}{\rm{D}}\times {\rm{T}}{\rm{C}}{\rm{C}})))$$

in which SR is the bulk sedimentation rate (cm year^−1^) using the age model of ref. ^15^, BD is the bulk density (g cm^−3^), TOM is the weight fraction total organic matter of the bulk sediment and TCC is the weight fraction CaCO_3_ of the bulk sediment. We calculated TOM (%) from TOC (%)/44 to reflect the molar ratio between plant cellulose (C_6_H_10_O_5_)*n* and TOC (%), and we calculated TCC (%) from TIC (%)/12 to reflect the molar ratio between TIC (%) and CaCO_3_. Because of the presence of both authigenic and detrital CaCO_3_ in the sediment core, the removal of all CaCO_3_ in the estimation of Flux_clastic_ results in an underestimation of the total detrital flux during intervals of high clastic input, and this, in turn, reduces the amplitude of change in clastic flux between intervals of high and low glacigenic sediment input. Although we do not explicitly remove the bSiO_2_ in the calculation of Flux_clastic_, the average weight percentage bSiO_2_ for all 65 facies samples is 0.92 ± 1.12% (±1 sigma), and—thus—is negligible. Because Flux_clastic_ is affected by sedimentation rates and because short-term changes in sedimentation rate require closely spaced age control, the highly variable Flux_clastic_ of the past 50 ka (Extended Data Fig. 2) compared with older intervals of the core is an artefact of having a much higher density of radiometric (radiocarbon) age control points over this interval. The more coarsely spaced U-Th and RPI tie points deeper in the core average out the extremes in sedimentation rate, thus dampening the range in Flux_clastic_.

### Palaeomagnetic polarity measurements

To provide a basal limiting date on the Junín record, four samples were obtained from core JUN15-1D-35H, the deepest core in the continuous stratigraphic splice^13^. Intervals were sampled with 8-cc discrete palaeomagnetic cubes centred at 93.80, 93.98, 94.65 and 94.78 m and measured at the Oregon State University (OR, USA) Paleo- and Environmental Magnetism Laboratory using a 2G Enterprises model 755-1.65UC superconducting rock magnetometer. Samples were demagnetized in an alternating field (AF) of 10, 15 and 20 mT to remove the relatively soft drilling overprint^15^ and show the primary direction recorded by a (post-)depositional remanent magnetization. Palaeomagnetic directions stabilized during demagnetization using the three peak AF fields following 20-mT AF demagnetization; inclination values of the four samples were −24.0°, −11.0°, +4.7° and −9.4°, respectively. These largely negative inclination values are consistent with, and/or are slightly shallower than, predicted inclination values for normal polarity of −21°, assuming a geocentric axial dipole field. Consistent relative declination values of 157°, 183°, 157° and 154°, respectively, suggest that the shallower inclination values deeper in the core reflect normal secular variation rather than variability driven by proximity to the Brunhes–Matuyama polarity reversal. As a result, it is probable that all four intervals were deposited during the Brunhes chron and are younger than 773 ka (ref. ^16^).

### Junín glaciation index

Downcore variations in MS and the ratio of the elements Ti to Ca are sensitive to the concentration of lithogenic material. Higher values of these parameters occur in glacial periods, related to the increased generation and delivery of clastic material to the lake during times when glaciers occupied the Lake Junín catchment. A compilation of sediment records from tropical Andean lakes from 2° S to 16° S (ref. ^8^) showed a close correspondence among the distribution of radiometric ages of moraines and variations in clastic sediment flux and concluded that the primary control on clastic sediment delivery to alpine lakes in the tropical Andes is the extent of glacial ice cover; basin-specific factors that might complicate this relationship, such as changes in sediment storage or paraglacial lag times, are not important on 10^4^-year timescales^8^. The absence of any evidence of notable clastic sediment delivery to Lake Junín during the current interglacial period (11,500 years ago to the present) suggests that precipitation events alone are unable to transport clastic sediment to the lake depocentre, and that it is the production of fine-grained glacial flour that is essential for the delivery of such sediment to the lake depocentre. Furthermore, fringing wetlands may prevent the small amounts of clastic sediment that may be transported from the Junín catchment from entering the lake today^12^ and, by extension, during previous interglacial intervals. The lithostratigraphy (Extended Data Fig. 1) is marked by repeated variations among a set of about six lithotypes, which implies that the lake’s sensitivity to record siliciclastic sediment did not change substantially over the past approximately 700 ka. To generate the Junín GI, we resampled both datasets on the radiometric and RPI age model^15^ at 250-year time steps. As both datasets were highly skewed, z-scores of the MS and Ti/Ca data were generated on the log-transformed data. The GI is the average z-score of both of these datasets, for which higher values indicate greater concentration of siliciclastic sediment in the cores. Comparison of the GI to an independent measure of lithogenic content is provided through the comparison with the log-transformed siliciclastic flux dataset (Extended Data Fig. 2) with a correlation (*r*) of 0.70.

### Time-series analysis

The GI dataset was selected for time-series analysis to examine the evolution of periodicities throughout the length of the record. Wavelet analysis^37^ of the 250-year-time-step GI dataset used a Morlet basis function in the PAST software package^48^, with the *p* = 0.05 significance level contoured in black (Fig. 3d).

## Online content

Any methods, additional references, Nature Research reporting summaries, source data, extended data, supplementary information, acknowledgements, peer review information; details of author contributions and competing interests; and statements of data and code availability are available at 10.1038/s41586-022-04873-0.

## Data Availability

All data will be archived at the NOAA National Centers for Environmental Information (www.ncei.noaa.gov/products/paleoclimatology). Source data are provided with this paper.

## Source data

Source Data Fig. 2
Source Data Fig. 3
Source Data Fig. 4
Source Data Extended Data Fig. 1
Source Data Extended Data Fig. 2
Source Data Extended Data Fig. 3
Source Data Extended Data Fig. 4

## References

[1] Woods A (2020). Andean drought and glacial retreat tied to Greenland warming during the last glacial period. Nat. Commun..

[2] Shackleton NJ, Opdyke ND (1973). Oxygen isotope and paleomagnetic stratigraphy of equatorial Pacific core V28-238: oxygen isotope temperatures and ice volumes on a 10^5^ year and 10^6^ year scale. Quat. Res..

[3] Lisiecki LE, Raymo ME (2005). A Pliocene-Pleistocene stack of 57 globally distributed benthic δ^18^O records. Paleoceanography.

[4] Denton, G. H. & Hughes, T. *The Last Great Ice Sheets* (Wiley, 1981).

[5] Ruddiman, W. F. *Earth*’*s Climate: Past and Future* (W.H. Freeman and Company, 2014).

[6] Smith JA, Seltzer GO, Farber DL, Rodbell DT, Finkel RC (2005). Early local last glacial maximum in the tropical Andes. Science.

[7] Putnam AE (2013). The Last Glacial Maximum at 44°S documented by a ^10^Be moraine chronology at Lake Ohau, Southern Alps of New Zealand. Quat. Sci. Rev..

[8] Rodbell DT, Seltzer GO, Mark BG, Smith JA, Abbott MB (2008). Clastic sediment flux to tropical Andean lakes: records of glaciation and soil erosion. Quat. Sci. Rev..

[9] Hooghiemstra H, Melice JL, Berger A, Shackleton NJ (1993). Frequency spectra and paleoclimatic variability of the high-resolution 30–1450 ka Funza I pollen record (Eastern Cordillera Colombia). Quat. Sci. Rev..

[10] Fritz SC (2007). Quaternary glaciation and hydrologic variation in the South American tropics as reconstructed from the Lake Titicaca drilling project. Quat. Res..

[11] Brook EJ, Sowers T, Orchardo J (1996). Rapid variations in atmospheric methane concentration during the past 110,000 years. Science.

[12] Seltzer G, Rodbell D, Burns S (2000). Isotopic evidence for late Quaternary climatic change in tropical South America. Geology.

[13] Hatfield RG (2020). Stratigraphic correlation and splice generation for sediments recovered from a large-lake drilling project: an example from Lake Junín, Peru. J. Paleolimnol..

[14] Chen CY (2020). U-Th dating of lake sediments: lessons from the 700 ka sediment record of Lake Junín, Peru. Quat. Sci. Rev..

[15] Hatfield RG (2020). Paleomagnetic constraint of the Brunhes age sedimentary record from Lake Junín, Peru. Front. Earth Sci..

[16] Channell JET, Hodell DA, Singer BS, Xuan C (2010). Reconciling astrochronological and 40Ar/39Ar ages for the Matuyama-Brunhes boundary and late Matuyama Chron. Geochem. Geophys. Geosyst..

[17] Smith JA, Seltzer GO, Rodbell DT, Klein AG (2005). Regional synthesis of last glacial maximum snowlines in the tropical Andes, South America. Quat. Int..

[18] Jouzel J (2007). Orbital and millennial Antarctic climate variability over the past 800,000 years. Science.

[19] Clark PU (2006). The middle Pleistocene transition: characteristics, mechanisms, and implications for long-term changes in atmospheric pCO_2_. Quat. Sci. Rev..

[20] Liu Y (2015). Obliquity pacing of the western Pacific Intertropical Convergence Zone over the past 282,000 years. Nat. Commun..

[21] Baker PA (2001). Tropical climate changes at millennial and orbital timescales on the Bolivian Altiplano. Nature.

[22] Shakun JD (2015). Regional and global forcing of glacier retreat during the last deglaciation. Nat. Commun..

[23] Stoll H (2020). 30 years of the iron hypothesis of ice ages. Nature.

[24] Thompson LG (1998). A 25,000-year tropical climate history from Bolivian ice cores. Science.

[25] Doughty AM, Kaplan MR, Peltier C, Barker SA (2021). A maximum in global glacier extent during MIS 4. Quat. Sci. Rev..

[26] Kanner LC, Burns SJ, Cheng H, Edwards RL (2012). High-latitude forcing of the South American summer monsoon during the last glacial. Science.

[27] Bond G (1993). Correlations between climate records from North Atlantic sediments and Greenland ice. Nature.

[28] Baker PA, Fritz SC (2015). Nature and causes of Quaternary climate variation of tropical South America. Quat. Sci. Rev..

[29] Sagredo E, Rupper S, Lowell T (2014). Sensitivities of the equilibrium line altitude to temperature and precipitation changes along the Andes. Quat. Res..

[30] Kaser G (2001). Glacier-climate interaction at low latitudes. J. Glaciol..

[31] Burns SJ, Welsh LK, Scroxton N, Cheng H, Edwards RL (2019). Millennial and orbital scale variability of the South American Monsoon during the penultimate glacial period. Sci. Rep..

[32] Barker S (2011). 800,000 years of abrupt climate variability. Science.

[33] Jomelli V (2014). A major advance of tropical Andean glaciers during the Antarctic cold reversal. Nature.

[34] He F (2013). Northern Hemisphere forcing of Southern Hemisphere climate during the last deglaciation. Nature.

[35] Kaser G, Juen I, Georges C, Gómez J, Tamayo W (2003). The impact of glaciers on the runoff and the reconstruction of mass balance history from hydrological data in the tropical Cordillera Blanca, Peru. J. Hydrol..

[36] Sobel AH, Bretherton CS (2000). Modeling tropical precipitation in a single column. J. Clim..

[37] Torrence C, Compo GP (1998). A practical guide to wavelet analysis. Bull. Am. Meteorol. Soc..

[38] Laskar J (2004). A long-term numerical solution for the insolation quantities of the Earth. Astron. Astrophys..

[39] Bereiter B (2015). Revision of the EPICA Dome C CO_2_ record from 800 to 600 kyr before present. Geophys. Res. Lett..

[40] Lüthi D (2008). High-resolution carbon dioxide concentration record 650,000–800,000 years before present. Nature.

[41] Loulergue L (2008). Orbital and millennial-scale features of atmospheric CH_4_ over the past 800,000 years. Nature.

[42] Instituto Geográfico Nacional (IGN). Ondores topographic map, scale 1:100,000. IGN, Lima, Perú (1968).

[43] Instituto Geográfico Nacional (IGN). Ulcumayo topographic map, scale 1:100,000. IGN, Lima, Perú (1985).

[44] Instituto Geográfico Nacional (IGN). Cerro de Pasco topographic map, scale 1:100,000. IGN, Lima, Perú (2000).

[45] Instituto Geográfico Nacional (IGN). Tarma topographic map, scale 1:100,000. IGN, Lima, Perú (2000).

[46] Rodbell, D. T., Abbott, M. B. & the 2011 ICDP Lake Junin Working Group, Workshop on drilling of Lake Junin, Peru: potential for development of a continuous tropical climate record. *Sci. Drill*. **13**, 58–60 (2012).

[47] DeMaster DJ (1981). The supply and accumulation of silica in the marine environment. Geochim. Cosmochim. Acta.

[48] Hammer Ø, Harper DAT, Ryan PD (2001). PAST: paleontological statistics software package for education and data analysis. Palaeontol. Electron..

[49] Lemieux-Dudon B (2010). Consistent dating for Antarctic and Greenland ice cores. Quat. Sci. Rev..

